# Comprehensive Genomic Assessment of Advanced-Stage GI Stromal Tumors Using the Japanese National Center for Cancer Genomics and Advanced Therapeutics Database

**DOI:** 10.1200/PO.24.00284

**Published:** 2024-10-24

**Authors:** Hiroyuki Fujii, Hidekazu Hirano, Kouya Shiraishi, Hirokazu Shoji, Toshiharu Hirose, Natsuko Okita, Atsuo Takashima, Takafumi Koyama, Ken Kato

**Affiliations:** ^1^ Department of Gastrointestinal Medical Oncology, National Cancer Center Hospital, Tokyo, Japan; ^2^ Department of Pulmonary Medicine, Graduate School of Medical Science, Kyoto, Japan; ^3^ Division of Genome Biology, National Cancer Center Research Institute, Tokyo, Japan; ^4^ Department of Experimental Therapeutics, National Cancer Center Hospital, Tokyo, Japan

## Abstract

**PURPOSE:**

Clinical utility of comprehensive genomic profiling (CGP) for precision medicine has become evident. Although there are several reports on the genomic landscape of GI stromal tumors (GISTs), large-scale data specific to GIST are limited, especially in Asia. Additionally, the applicability of molecular-targeted agents identified using CGP has not been extensively examined. We investigated the status of genomic alterations in Japanese patients with advanced GISTs using the National Center for Cancer Genomics and Advanced Therapeutics (C-CAT) database to identify novel treatment strategies and drug development.

**MATERIALS AND METHODS:**

We retrospectively reviewed the clinical and CGP data of patients with advanced-stage GIST registered in the C-CAT database to assess the genomic landscape and potential actionable alterations.

**RESULTS:**

Data from 144 patients were reviewed. Oncogenic alterations were detected frequently in *KIT* (78%), *CDKN2A* (37%), *CDKN2B* (29%), *RB1* (11%), *STK11* (10%), *TP53* (9%), *PDGFRA* (6%), and *SDHB* (6%). Loss of *CDKN2A*/*CDKN2B* was only observed in *KIT/PDGFRA*-mutated GISTs, while alterations in *SDHA/SDHB* were only detected in *KIT/PDGFRA* wild-type GISTs. Among 119 *KIT/PDGFRA*-mutated GISTs, 95 (80%) had oncogenic genomic alterations and 29 (24%) had actionable alterations, excluding *KIT* and *PDGFRA*. However, among 25 *KIT/PDGFRA* wild-type GISTs, 22 (88%) had oncogenic alterations and 11 (44%) had actionable alterations. Representative candidate drugs for genome-matched therapies in *KIT/PDGFRA*-mutated and wild-type GISTs were as follows: pembrolizumab for tumor mutation burden–high in one and two patients, respectively; poly-adenosine diphosphate ribose polymerase inhibitors for alterations related to homologous recombination deficiency in 12 and one patient, respectively; NTRK inhibitor for *ETV6-NTRK3* fusion in one with *KIT/PDGFRA* wild-type GIST; and human epidermal growth factor receptor 2-antibody-drug conjugate in one with *KIT/PDGFRA*-mutated GIST.

**CONCLUSION:**

This study highlights the genomic landscape of advanced GISTs and the important role of CGP in identifying rational molecular-targeted therapeutic options.

## Teaser

Genomic landscape of advanced GISTs reveals actionable alterations, guiding targeted therapy using the C-CAT database.

## INTRODUCTION

GI stromal tumors (GISTs) are sarcomas derived from the precursors of the interstitial cells of Cajal, and the most common mesenchymal tumors of the GI tract, with an annual incidence of 6-22 per million people and an estimated prevalence of 129 per million.^1-3^

**CONTEXT**

**Key Objective**
There is room for further investigation into the genomic landscape analysis using comprehensive genomic profiling (CGP) in advanced GI stromal tumors (GISTs) and the applicability of the identified molecular-targeted agents. This study aimed to analyze the genomic landscape of advanced GISTs using CGP data from the National Center for Cancer Genomics and Advanced Therapeutics database to evaluate and characterize the genomic alterations that could potentially be therapeutic targets in GISTs.
**Knowledge Generated**
Genomic status and therapeutic target gene alterations differed between *KIT/PDGFRA*-mutated and *KIT/PDGFRA* wild-type GISTs. Twenty-eight percent of patients had actionable alterations that could be therapeutic targets of existing approved drugs or off-label drugs.
**Relevance**
These findings shed light on the potential for personalized treatment strategies in GISTs, suggesting the importance of CGP in guiding therapeutic decisions and the development of molecular-targeted therapies. The study provides valuable insights for clinicians and researchers aiming to optimize treatment approaches for patients with GIST.

With advances in molecular biology, the activated molecular pathways associated with the pathogenicity of GISTs have been identified. The most frequent driver mutations occur in *KIT* (60%-70%) and *PDGFRA* (10%-15%).^2,4-7^ GISTs are typically classified as either *KIT/PDGFRA*-mutated or *KIT/PDGFRA* wild-type because they have distinct clinical characteristics and behaviors, such as sensitivity to imatinib. *KIT/PDGFRA* wild-type GISTs lack growth-stimulating mutations, rendering tyrosine kinase inhibitors relatively ineffective. *KIT/PDGFRA* wild-type GISTs are characterized by *SDH* dysfunction or other less frequent alterations, such as somatic mutations in *NF1* or *BRAF*, and fusion in *NTRK3* or *FGFR1*.^8-11^ However, we should note that these reports were mainly for primarily localized GISTs.

Comprehensive genomic profiling (CGP) using next-generation sequencing (NGS), which can analyze multiple genomic alterations simultaneously, plays an important role in advancing precision medicine in oncology.^12,13^ Two reports on the CGP of soft tissue and bone sarcoma were simultaneously published by researchers from the Memorial Sloan Kettering Cancer Center. Gounder et al^14^ analyzed the genomic landscape of 7,494 patients across 44 different sarcoma subtypes, and Nacev et al^15^ characterized genomic alterations in a large cohort of 2,138 sarcomas encompassing 45 subtypes, with GISTs representing 104 (1.4%) and 395 (18.5%) cases, respectively. However, while the main focus was on comparing the genomic landscape across different numerous sarcoma subtypes, specific analyses dedicated exclusively to GISTs were limited. This fact leaves room for further discussion on the genomic characteristics of advanced GISTs and how precision medicine can enhance treatment outcomes for these tumors. Despite the availability of several drugs after resistance to imatinib has developed, their effectiveness is limited. For advanced GISTs with poor prognosis, it is worthwhile to explore the potential of leveraging treatments developed for other types of cancers and to identify new drug targets on the basis of CGP.

Therefore, we investigated the status of genomic alterations in patients with advanced GISTs who required molecular-targeted therapy using the National Center for Cancer Genomics and Advanced Therapeutics (C-CAT; Tokyo, Japan) database to identify novel strategies for clinical treatment and early drug development. The primary focus was to identify the genomic landscape and evaluate the frequency of oncogenic and actionable genomic alterations that could potentially be therapeutic targets in advanced GISTs. Additionally, although molecular targeted therapies are applied, current treatment strategies for advanced GISTs are uniform and do not vary on the basis of genomic backgrounds except for *PDGFRA* D842V mutation. A secondary focus involved comparing the characteristics of oncogenic and actionable genomic alterations by classifying GISTs into *KIT/PDGFRA*-mutated and *KIT/PDGFRA* wild-type subgroups. We also examined whether the genomic profiles differ between patients who had samples taken before imatinib treatment and those taken after, to explore temporal genomic variations.

## MATERIALS AND METHODS

We retrospectively reviewed the data of patients with advanced GIST entered in the C-CAT database from October 2020 to April 2023.^16^ The C-CAT database inclusively aggregates clinical and genomic information on patients who underwent CGP under the Japanese national health insurance system. We used two different NGS test platforms: OncoGuide NCC Oncopanel System (NCC Oncopanel)^17^ and FoundationOne CDx Cancer Genomic Profile (F1).^18^ We obtained the clinical data and CGP data from the C-CAT database (ver. 6.0.0). CGP data included genomic alterations, annotated clinical significance, microsatellite instability (MSI), tumor mutation burden (TMB), and information on drugs expected to be effective against these genomic alterations. Clinical characteristics were those at the time of CGP. Data cutoff was set for May 2023. We accessed the C-CAT data through the C-CAT Research-Use Portal site after obtaining approval from the National Cancer Center-Institutional Review Board (approval number 2020-067) and C-CAT Data Utilization Review Board (approval number CDU2021-001N).

### Selection of Candidate Variants and Drugs for Oncogenic Genomic Alterations

We annotated the NGS results using C-CAT proprietary algorithms,^16^ Association for Molecular Pathology, ASCO, and College of American Pathologists joint guidelines,^19^ and public databases (COSMIC,^20^ ClinVar,^21^ and CIViC^22^). In this study, small-scale variants included single-nucleotide variants and insertion/deletions. We defined copy-number variation (CNV) when the copy-number ratio of the region in the detected gene was ≤1/4 (loss) or ≥4 (amplification). Multiple classes of alterations refer to the simultaneous combination of small-scale variants and CNV in the same gene. We annotated genomic alterations broadly into six categories: pathogenic (oncogenic), likely pathogenic (oncogenic), variant of unknown significance, likely benign, benign, and unknown, with reference mainly to CIViC. We defined TMB-high as ≥10 mutations/megabase (Muts/Mb). We classified genomic alterations annotated as pathogenic (oncogenic) or likely pathogenic (oncogenic) as oncogenic alterations associated with disease progression in GISTs.

We also classified actionable alterations as oncogenic alterations excluding *KIT* and *PDGFRA* that could be treated by the administration of existing approved drugs for pan-cancer or off-label drugs (approved for other cancer types).

### Statistical Analysis

We used Fisher's exact test or the chi-square test for categorical data and Student's *t*-test for continuous data to compare differences in the detection rate of genomic alterations and characteristics between groups. Statistical tests were two-sided, and a *P* < .05 was considered statistically significant. All statistical analyses were performed using the R-statistical software (The R-Foundation for Statistical Computing, Vienna, Austria).

## RESULTS

### Patient Characteristics

Up to April 2023, a total of 144 patients with advanced GISTs were enrolled into the C-CAT database; their baseline clinicopathologic characteristics are summarized in Table 1. Eight patients were assessed with NCC Oncopanel (6%) and 136 patients were assessed with F1 (94%). Fifty-four patients (38%) underwent CGP using specimens obtained before imatinib treatment (before group) and 90 (63%) using specimens obtained after imatinib treatment (after group).

**TABLE 1.** Patient Characteristics

Characteristic	All Patients (N = 144)
Age, years, median (range)	61 (19-87)
Age <60 years, No. (%)	68 (47)
Age ≥60 years, No. (%)	76 (53)
Sex, No. (%)	
Male	76 (53)
Female	68 (47)
ECOG-PS, No. (%)	
0	101 (70)
1-2	43 (30)
Primary site, No. (%)	
Stomach	50 (35)
Small intestine	46 (32)
Colon	5 (4)
Rectum	2 (1)
Unknown	41 (29)
Clinical staging, No. (%)	
Recurrent	50 (35)
Unresectable	94 (65)
Metastatic organ, No. (%)	
Liver	77 (54)
Peritoneum	75 (52)
Lymph node	14 (10)
Lung/pleura	8 (6)
Bone	6 (4)
CGP test platform, No. (%)	
NCC Oncopanel	8 (6)
F1	136 (94)
Sampling site, No. (%)	
Primary site	68 (47)
Metastatic site	76 (53)
Sampling timing, No. (%)	
Before imatinib	54 (38)
After imatinib	90 (63)

Abbreviations: CGP, comprehensive genomic profiling; ECOG-PS, Eastern Cooperative Oncology Group Performance Status; F1, FoundationOne CDx Cancer Genomic Profile; NCC Oncopanel, OncoGuide NCC Oncopanel System.

### Oncogenic Genomic Alterations of Advanced GISTs

**FIG 1.**
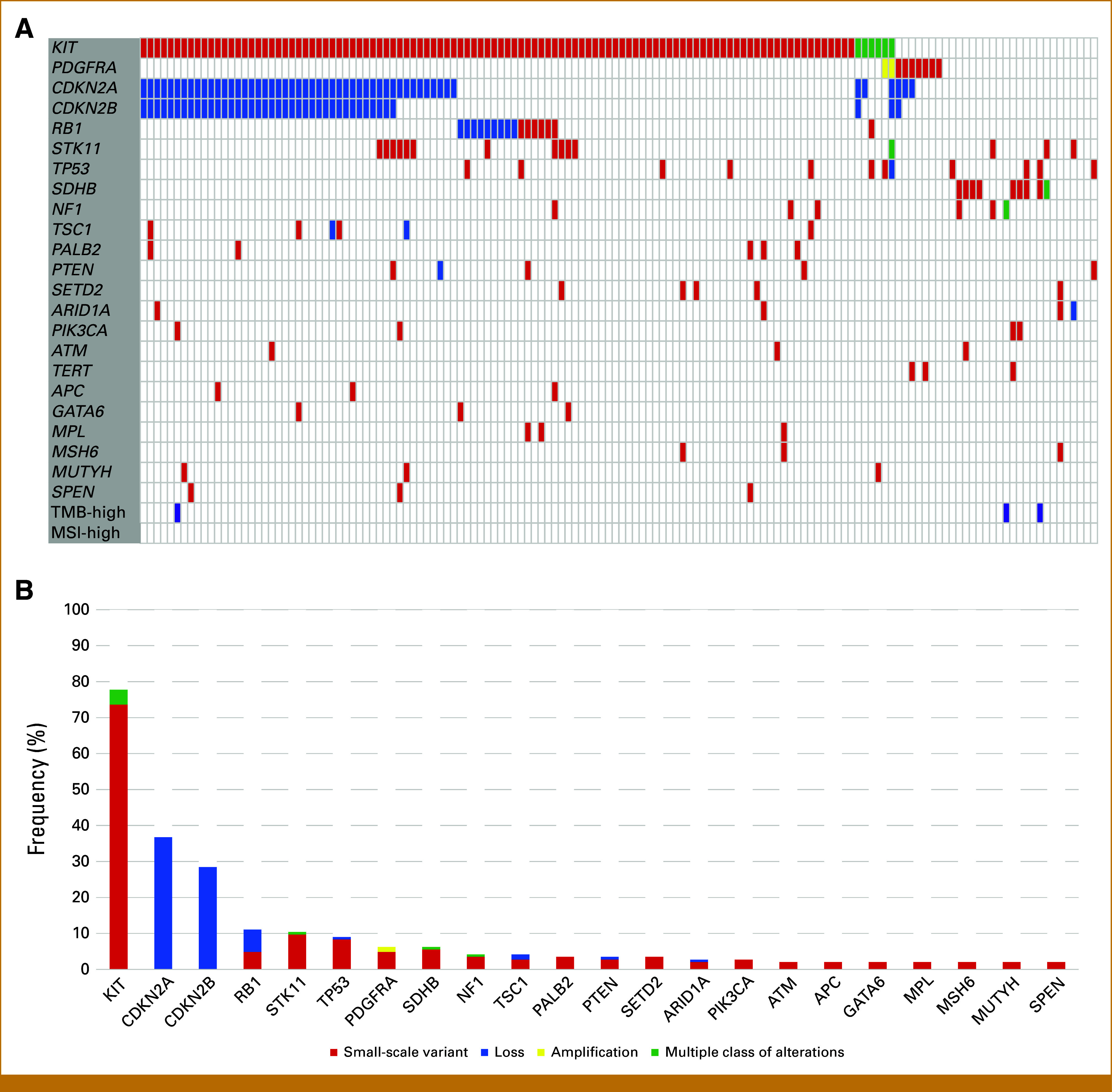
(A) Landscape and (B) bar graph of oncogenic genomic alterations in specific genes detected at a frequency >2% and TMB-high and MSI-high status. MSI, microsatellite instability; TMB, tumor mutation burden.

**FIG A1.**
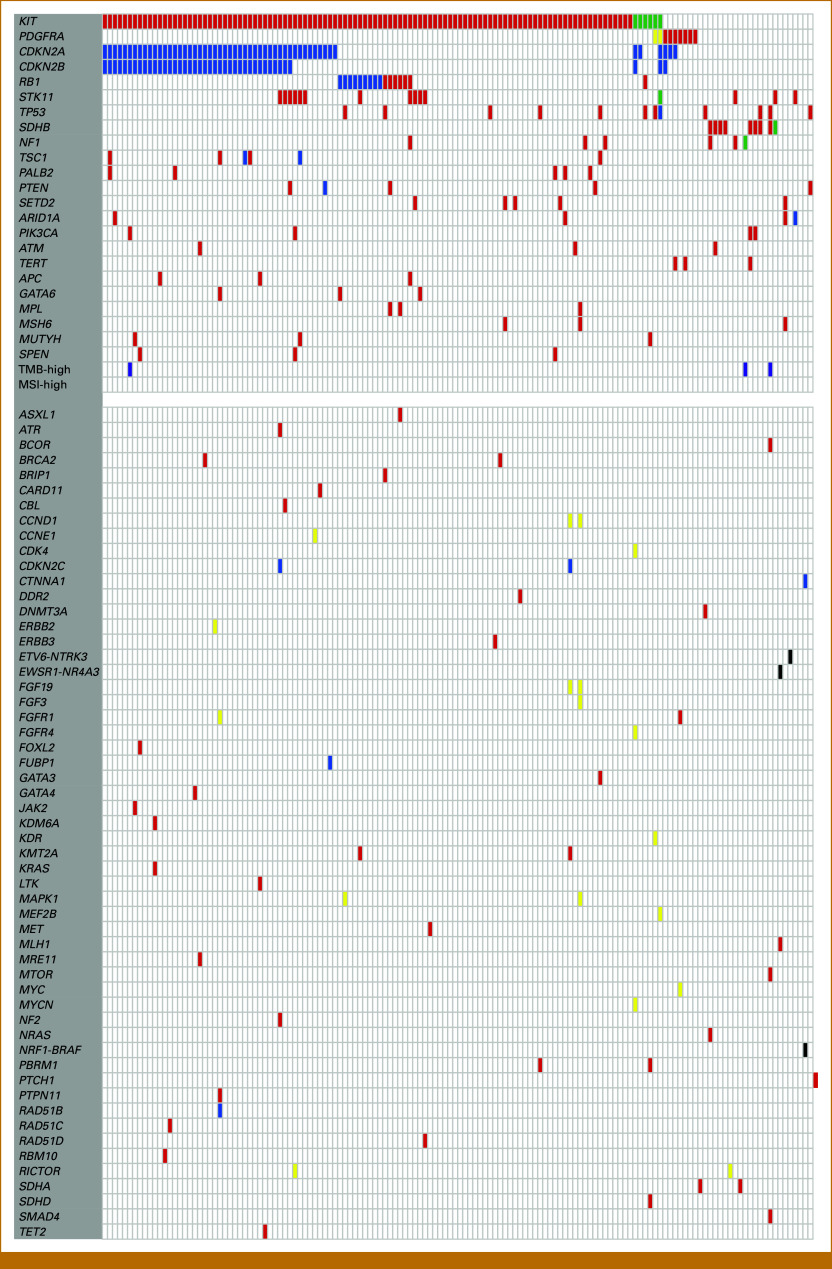
Landscape of overall oncogenic genomic alterations and TMB-high and MSI-high status. Red bar: small-scale variant; blue bar: loss; yellow bar: amplification; green bar: multiple class of alterations; purple bar: TMB-high; black bar: fusion. MSI, microsatellite instability; TMB, tumor mutation burden.

Oncogenic genomic alterations in specific genes detected at a frequency >2% in 144 patients and TMB-high and MSI-high status are described in Figures 1A and 1B. Overall genomic alterations with MSI and TMB status are shown in Appendix Figure A1. The most frequently observed oncogenic genomic alterations were in *KIT* (78%), *CDKN2A* (37%), *CDKN2B* (29%), *RB1* (11%), *STK11* (10%), *TP53* (9%), *PDGFRA* (6%), *SDHB* (6%), and *NF1* (4%). No patients had the *BRAF* V600E mutation. No patients had MSI-high status; however, three had TMB-high status (2%) and one had *ETV6-NTRK3* fusion (1%).

A total of 119 patients (83%) had *KIT/PDGFRA*-mutated GISTs, whereas 25 (17%) had *KIT/PDGFRA* wild-type GISTs. The small-scale variants of *KIT* and *PDGFRA* were mutually exclusive, but two patients had *KIT* and *PDGFRA* amplification simultaneously. These two patients underwent sampling biopsy for CGP after treatment with imatinib. The *PDGFRA* D842V mutation was found in four patients (3%). Clinicopathologic characteristics are compared between both groups in Appendix Table A1. *KIT/PDGFRA* wild-type GISTs were more likely to include younger and more female patients. And also, *KIT/PDGFRA* wild-type GISTs had significantly more primary gastric tumors and metastases to the lymph nodes than patients with *KIT/PDGFRA*-mutated GISTs, which commonly metastasized to the liver and peritoneum.

### Differences in Oncogenic Genomic Alterations in *KIT/PDGFRA*-Mutated GISTs and *KIT/PDGFRA* Wild-Type GISTs

**FIG 2.**
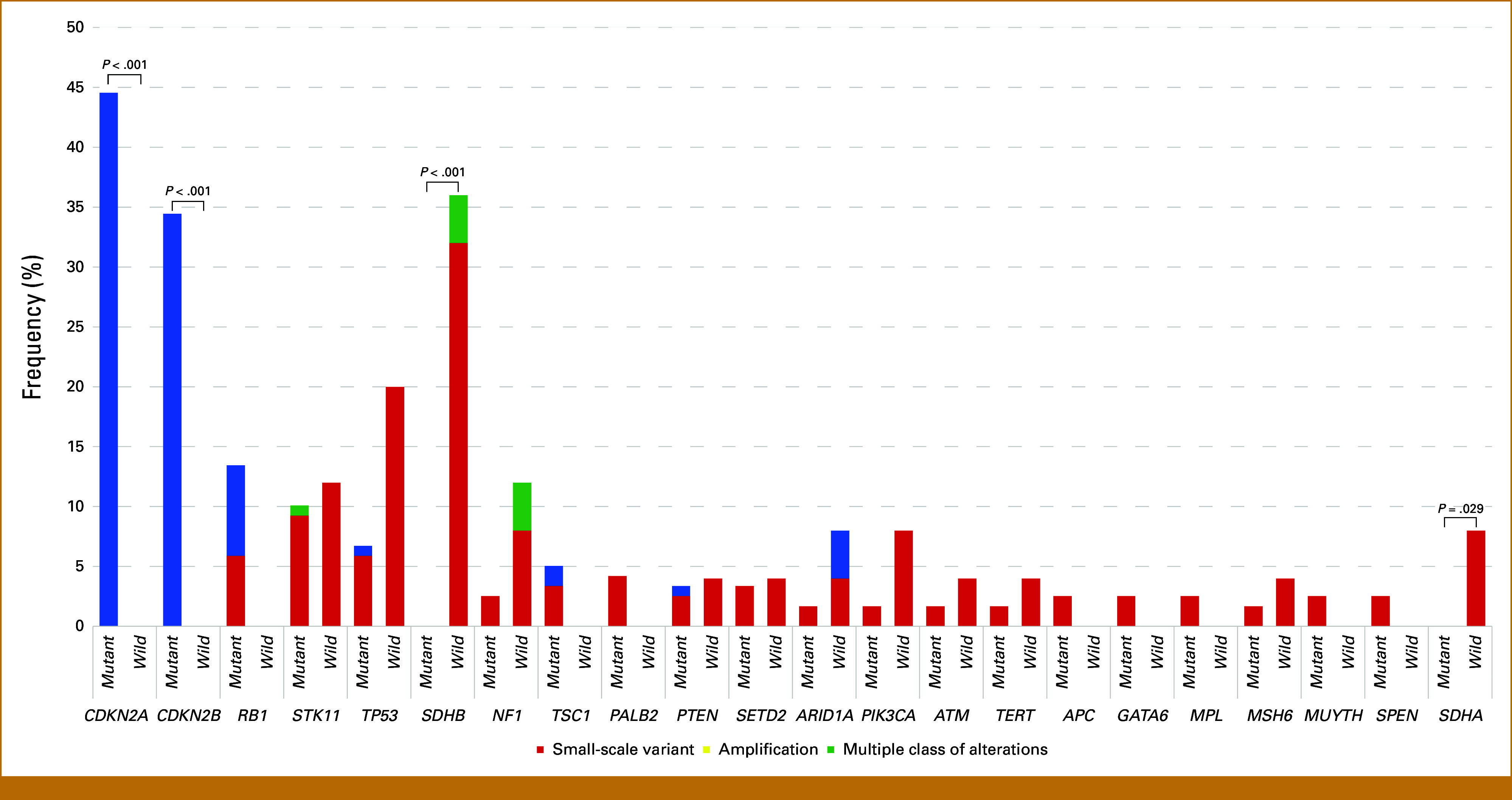
Comparison of oncogenic genomic alterations, excluding *KIT* and *PDGFRA*, in *KIT/PDGFRA-*mutated GISTs (n = 119; mutant) and *KIT/PDGFRA* wild-type GISTs (n = 25; wild). The bar graph indicates oncogenic genomic alterations in specific genes detected at a frequency >2%. *P* values are noted for genes with a significant difference in alteration frequency between the two types. GISTs, GI stromal tumors.

**FIG A2.**
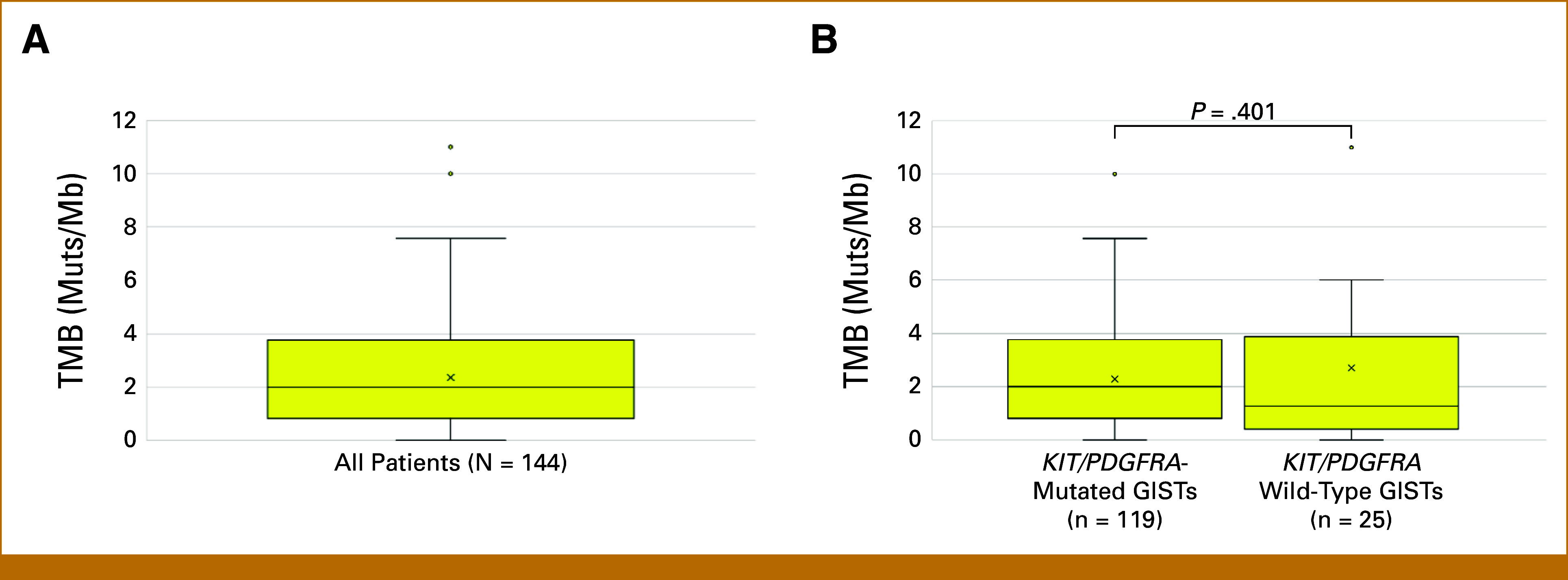
(A) Distribution of TMB levels in all patients. (B) Comparison of TMB levels between *KIT/PDGFRA-*mutated GISTs and *KIT/PDGFRA* wild-type GISTs. GISTs, GI stromal tumors; Muts/Mb, mutations/megabase; TMB, tumor mutation burden.

Oncogenic genomic alterations, excluding *KIT* and *PDGFRA,* were detected in 95 patients (80%) with *KIT/PDGFRA*-mutated GISTs and 22 (88%) with *KIT/PDGFRA* wild-type GISTs. Figure 2 shows the comparison of oncogenic genomic alterations, excluding *KIT* and *PDGFRA,* detected at a frequency >2% in each group. In *KIT/PDGFRA*-mutated GISTs, the most common oncogenic genomic alterations, excluding *KIT* and *PDGFRA,* were *CDKN2A* (45%), *CDKN2B* (35%), *RB1* (13%), *STK11* (9%), and *TP53* (7%). In *KIT/PDGFRA* wild-type GISTs, *SDHB* (36%), *TP53* (20%), *NF1* (12%), *STK11* (12%), and *SDHA* (8%) were the most common oncogenic genomic alterations with *ETV6-NTRK3* fusion detected in one patient (2%). Loss of *CDKN2A* and *CDKN2B* was observed only in *KIT/PDGFRA*-mutated GISTs and the detection frequencies between both groups were significantly different (*P* < .001). Oncogenic coalterations in *RB1*, *TSC1*, *PALB2*, *APC*, *GATA6*, *MPL*, and *SPEN* were observed only in *KIT/PDGFRA*-mutated GISTs. Alterations in *SDHB* and *SDHA* were detected only in *KIT/PDGFRA* wild-type GISTs (*P* < .001 and *P* = .029, respectively). There was no difference in TMB between *KIT/PDGFRA*-mutated GISTs and *KIT/PDGFRA* wild-type GISTs (average, 2.29 *v* 2.71 Muts/Mb; *P* = .401), and there were one and two TMB-high patients in each group, respectively (Appendix Figs A2A and A2B).

### Comparison of Detected Oncogenic Genomic Alterations Depending on Sampling Timing

**FIG 3.**
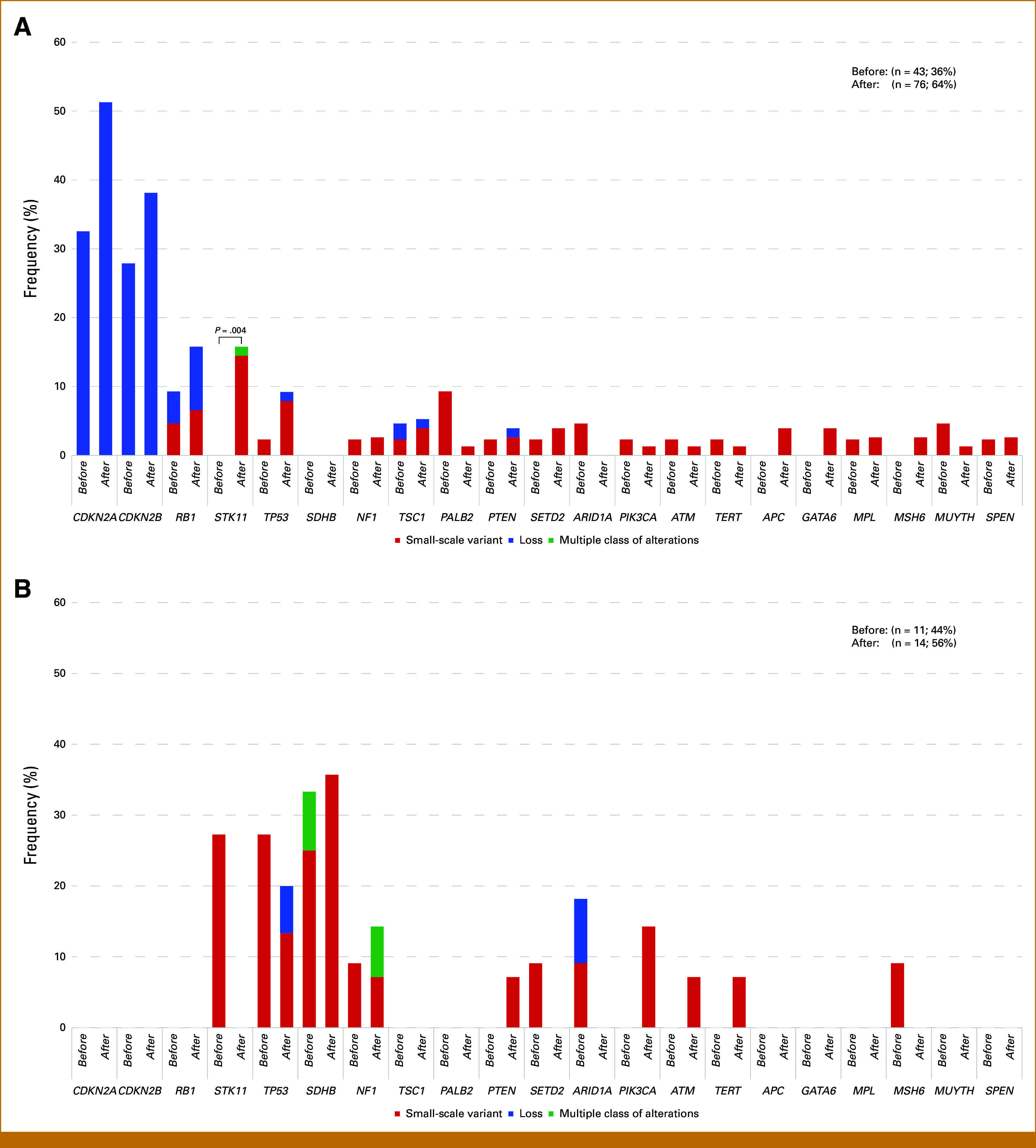
Comparison of detected oncogenic genomic alterations by dividing sampling timing of the specimen for CGP into before imatinib treatment (before group) and after imatinib treatment (after group). Bar graph of the variant status of specific genes detected at a frequency >2% in the entire population. *P* values are noted for genes with a significant difference in alteration frequency between the two groups. (A) *KIT/PDGFRA-*mutated GISTs. (B) *KIT/PDGFRA* wild-type GISTs. CGP, comprehensive genomic profiling; GISTs, GI stromal tumors.

Oncogenic genomic alterations were compared in patients with *KIT/PDGFRA*-mutated GISTs or *KIT/PDGFRA* wild-type GISTs who underwent CGP using the before and after groups. Among *KIT/PDGFRA*-mutated GISTs, the *STK11* variant was detected more frequently in the after group than in the before group (16% *v* 0%; *P* = .004), but there was no trend in *KIT/PDGFRA* wild-type GISTs (Figs 3A and 3B).

**FIG A3.**
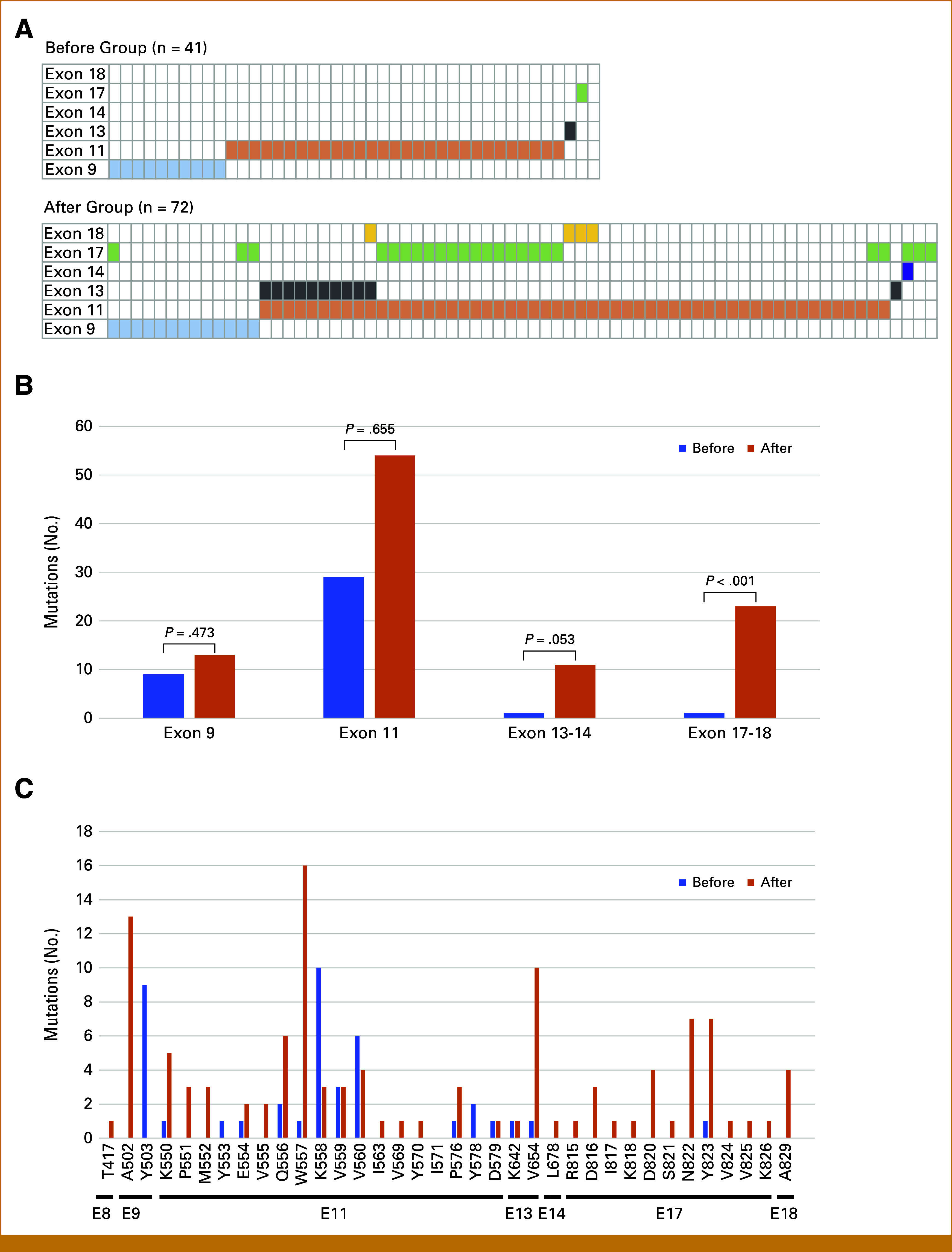
(A) Mutation plot of *KIT* in each exon in the before (n = 41) and after (n = 72) groups. (B) Comparison of detected *KIT* mutations in each exon in the before and after groups. (C) Details of the *KIT*-affected exons and codons.

As for *KIT* in *KIT/PDGFRA*-mutated GISTs, mutations within each exon were compared between the before and after groups. Appendix Figure A3A shows a mutation plot for each exon in both groups. In the before group, all patients had only one mutation in *KIT*: nine (22%) in exon 9; 29 (71%) in exon 11; and one (2%) in exons 13 and 17. By contrast, 35 patients (49%) in the after group had two or more KIT mutations in separate exons. Mutations in exons 14 and 18, which were not detected in the before group, were detected in one (1%) and four patients (6%), respectively, in the after group. There was no difference in the frequency of mutations in exons 9 and 11 between the two groups. However, mutations in exons 13-14 (adenosine triphosphate [ATP]–binding domain; 15% *v* 2%, respectively; *P* = .053) and exons 17-18 (kinase loop domain; 32% *v* 2%, respectively; *P* < .001) were detected more frequently in the after group than in the before group (Appendix Fig A3B). In the after group, the most common *KIT* mutations in exons 13-14 or 17-18 were V654 in exon 13 (14%), N822 in exon 17 (10%), Y823 in exon 17 (10%), D820 in exon 17 (6%), and A829 in exon 18 (6%; Appendix Fig A3C).

### Differences in Actionable Genomic Alterations and Corresponding Candidate Drugs in *KIT/PDGFRA*-Mutated GISTs and *KIT/PDGFRA* Wild-Type GISTs

**FIG 4.**
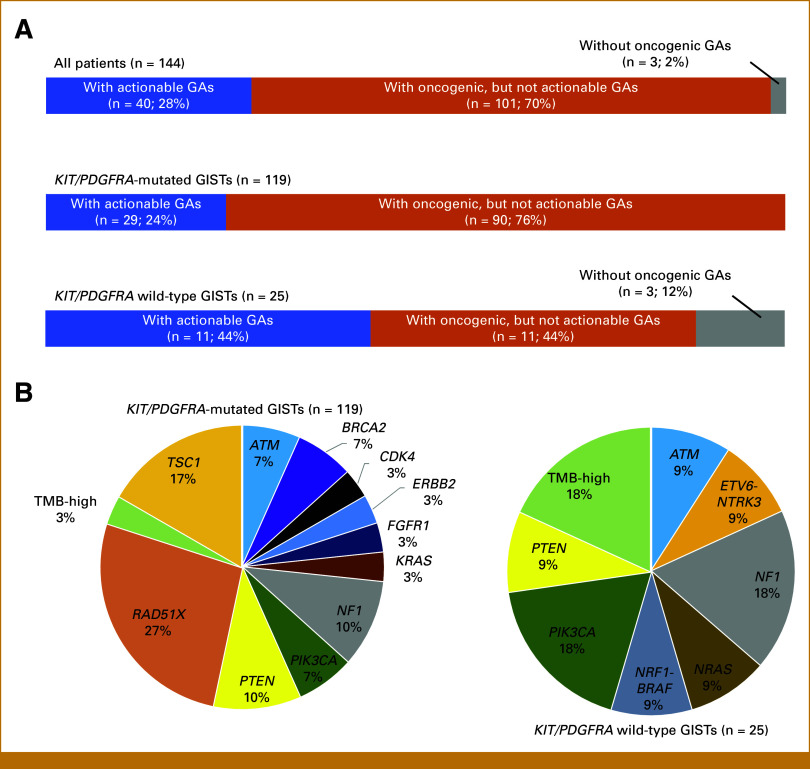
(A) Breakdown of the proportion of patients with actionable GAs; with oncogenic, but not actionable GAs; and without oncogenic GAs in *KIT/PDGFRA-*mutated GISTs (n = 119) and *KIT/PDGFRA* wild-type GISTs (n = 25). (B) Breakdown of the evidence level for actionable GAs and drugs for level A in each of the *KIT/PDGFRA-*mutated GISTs and *KIT/PDGFRA* wild-type GISTs. GAs, genomic alterations; GISTs, GI stromal tumors.

Of the 119 patients with *KIT/PDGFRA*-mutated GISTs, 29 (24%) had actionable genomic alterations. Of the 25 patients with *KIT/PDGFRA* wild-type GISTs, 11 (44%) had actionable genomic alterations (Fig 4A). Figure 4B and Table 2 show the breakdown of the actionable genomic alterations and corresponding candidate drugs. In *KIT/PDGFRA*-mutated GISTs, one patient with TMB-high was proposed for treatment with pembrolizumab. For *KIT/PDGFRA* wild-type GISTs, three patients were proposed for treatment with approved drugs: two TMB-high patients for pembrolizumab and one patient with *ETV6-NTRK3* fusion for entrectinib or larotrectinib.

**TABLE 2.** Combination of Specific Actionable Genomic Alterations and Candidate Therapeutic Drugs

Gene	Type of Genomic Alterations	Candidate Drug	Drug Status	*KIT/PDGFRA-*Mutated GISTs (n = 119), No. (%)	*KIT/PDGFRA* Wild-Type GISTs (n = 25), No. (%)
*ATM*	Small-scale variant	Olaparib	Off-label use	2 (2)	1 (4)
*BRCA2*	Small-scale variant	Olaparib	Off-label use	2 (2)	0
*CDK4*	Amplification	Palbociclib	Off-label use	1 (1)	0
*ETV6-NTRK3*	Fusion	Entrectinib	Approved	0	1 (4)
*ERBB2*	Amplification	Trastuzumab-deruxtecan	Off-label use	1 (1)	0
*FGFR1*	Amplification	Infigratinib	Off-label use	1 (1)	0
*KRAS* G12C	Small-scale variant	Sotorasib	Off-label use	1 (1)	0
*NF1*	Small-scale variant	Selumetinib	Off-label use	3 (3)	2 (8)
*NRAS*	Small-scale variant	Cobimetinib	Off-label use	0	1 (4)
*NRF1-BRAF*	Fusion	Cobimetinib	Off-label use	0	1 (4)
*PIK3CA*	Small-scale variant	Alpelisib	Off-label use	2 (2)	2 (8)
*PTEN*	Small-scale variant Loss	Everolimus	Off-label use	3 (3)	1 (4)
*RAD51X*	Loss Small-scale variant	Olaparib	Off-label use	8 (7)	0
*TSC1*	Small-scale variant Loss	Everolimus	Off-label use	5 (4)	0
TMB-high	Other	Pembrolizumab	Approved	1 (1)	2 (8)

Abbreviations: GISTs, GI stromal tumors; TMB, tumor mutation burden.

Genomically matched therapy excluding approved drugs (off-label use) comprised MEK inhibitors, FGFR inhibitor, PIK3CA inhibitor, KRAS inhibitor, mammalian target of rapamycin (mTOR) inhibitor, antibody-drug conjugates (ADCs), poly-adenosine diphosphate ribose polymerase (PARP) inhibitor, and CDK4/6 inhibitor. MEK inhibitors included selumetinib for small-scale variants of *NF1* and cobimetinib for small-scale variants of *NRAS* and *BRAF* fusion. The potential of infigratinib for *FGFR1* amplification and alpelisib for small-scale variants of *PIK3CA* was suggested. Sotorasib for *KRAS* G12C-mutated tumors was proposed. Everolimus was identified as a candidate for genetic abnormalities associated with the mTOR pathway (loss of function of *TSC1* and *PTEN*). Regarding ADC, trastuzumab-deruxtecan was proposed for one *KIT/PDGFRA*-mutated GIST patient with *ERBB2* amplification. In addition, the proportion of patients harboring genomic alterations associated with homologous recombination deficiency (small-scale variant or loss of *ATM*, *BRCA2*, and *RAD51X*) was higher in *KIT/PDGFRA*-mutated GISTs than in *KIT/PDGFRA* wild-type GISTs (10% *v* 4%, respectively; *P* = .467). PARP inhibitors, such as olaparib, are potential treatments for these patients.

## DISCUSSION

To our knowledge, this study reports the largest CGP analysis of Japanese patients with advanced GISTs showing the prevalence and spectrum of oncogenic gene alterations using the C-CAT database. Also, our study elucidated the landscape of genomic alterations in advanced GISTs and demonstrated disparities in genomic alterations between *KIT/PDGFRA*-mutated GISTs and *KIT/PDGFRA* wild-type GISTs.

Our study found that the prevalence and clinical profiles of patients with *KIT/PDGFRA*-mutated GISTs and *KIT/PDGFRA* wild-type GISTs were consistent with those reported previously.^2,4-11^ Gastric primary tumors, female patients, younger patients, and lymph node metastases were more frequent in *KIT/PDGFRA* wild-type GISTs than in *KIT/PDGFRA* mutant GISTs. These features are known to be common in SDH-deficient GISTs and could be explained by the high prevalence of *KIT/PDGFRA* wild-type GIST patients with a genomic alteration in *SDHX*. It was valuable to confirm the similarities in the genomic backgrounds of GISTs in Asian and Western countries using CGP analysis.

In the Japanese population, 3% of patients with GIST harbored the *PDGFRA* D842V mutation, which is characterized by a poor response to imatinib, sunitinib, and regorafenib.^23-25^ In the NAVIGATOR phase I trial, avapritinib demonstrated an impressive overall response rate (88%) for *PDGFRA* D842V-mutated GISTs, leading to its approval by the US Food and Drug Administration.^26^ However, avapritinib is not approved in many countries, including Japan. Therefore, clinical development in unapproved countries is essential to improve treatment outcomes in patients with *PDGFRA* D842V-mutated GISTs.

In agreement with previous reports, post-imatinib samples were associated with a higher frequency of *KIT* mutations in the ATP-binding pocket (exons 13-14) or kinase loop domains (exons 17-18).^24^ The efficacy of sunitinib may differ according to the location of *KIT* secondary mutations.^27^ This implies that CGP of post-imatinib samples may help identify a subset of patients who are likely to have a favorable response to sunitinib. *STK11* was also detected significantly more frequently in post-imatinib samples of *KIT/PDGFRA*-mutated GISTs. *STK11* alterations are associated with poor clinical outcomes in non–small cell lung cancer (NSCLC),^28^ suggesting that the loss of *STK11* function may be involved in imatinib resistance. Furthermore, the patients who exhibited simultaneous amplification of both *KIT* and *PDGFRA* had undergone imatinib treatment before tissue sampling. It has been suggested that *KIT* amplification alone is unlikely to significantly contribute to acquired resistance to imatinib,^29^ but the coexistence of *PDGFRA* amplification might be leading to the development of resistance.

We elucidated the in-depth genomic profiles for the two GIST subtypes. *KIT/PDGFRA*-mutated GISTs were associated with a high prevalence of *CDKN2A/B* and *RB1* loss. These molecular dysregulations are thought to facilitate the cell cycle and explain the malignant transformation of GISTs.^30^ By contrast, no *KIT/PDGFRA* wild-type GISTs exhibited *CDKN2A/B* or *RB1* loss. GISTs with an *SDHA/B* alteration, which account for the majority of wild-type GISTs, progress because of the dysfunction of the SDH complex involved in the mitochondrial tricarboxylic acid cycle and the activation of hypoxia-inducible factor (HIF)-1/2, which drives further angiogenesis, cell growth, and tumorigenic transformation.^31^ The prevalence of *RB* alterations in *KIT/PDGFRA*-mutated GISTs may be due to the role of underlying cell cycle abnormalities in pathogenesis. Our results suggest that the pathogenesis of *KIT/PDGFRA* wild-type GISTs differs significantly from that of *KIT/PDGFRA*-mutated GISTs on the basis of the genomic landscape.

Most major clinical trials have treated advanced GISTs as a single disease. Strategies for the treatment and drug development of advanced GISTs on the basis of their genomic profiles may improve the clinical outcomes of patients with GIST, as in NSCLC. On the basis of our analysis, we expect that combination therapy with CDK4/6 and CDK2 inhibitors, which is currently under development, will be effective for *KIT/PDGFRA*-mutated GISTs with *CDKN2A/B* coalteration.^32^ By contrast, HIF-2α inhibitors, such as belzutifan, may be good candidates for *KIT/PDGFRA* wild-type GISTs with *SDH* alteration (ClinicalTrials.gov identifier: NCT04924075).

The identification of actionable genomic alterations in 24% of *KIT/PDGFRA-*mutated GISTs and 44% of *KIT/PDGFRA* wild-type GISTs holds significant promise for expanding treatment options. As CGP can serve to increase the therapeutic options linked to actionable genomic alterations, more studies are needed to verify whether genomically matched treatment is truly efficacious for advanced-stage GISTs.

As a limitation of this study, we did not evaluate the efficacy of genome-matched therapy because of the limited availability of the recommended treatments.

In conclusion, we determined the genomic profiles of patients with advanced GISTs using the C-CAT database. Various genomic statuses were highlighted, depending on *KIT/PDGFRA-*mutated GISTs or *KIT/PDGFRA* wild-type GISTs, and whether the specimens were obtained before or after imatinib administration. As the development of precision medicine continues to advance dramatically, we hope that our results provide some insights into the treatment of GISTs.

## APPENDIX

**TABLE A1.** Comparison of Clinical Characteristics in *KIT*/*PDGFRA*-Mutated GISTs and *KIT*/*PDGFRA* Wild-Type GISTs

Characteristic	*KIT*/*PDGFRA*-Mutated GISTs (n = 119)	*KIT*/*PDGFRA* Wild-Type GISTs (n = 25)	*P*
Age, years, median (range)	64 (31-87)	45 (19-71)	<.001
Age <60 years, No. (%)	50 (42)	18 (72)	
Age ≥60 years, No. (%)	69 (58)	7 (28)	
Sex, No. (%)			.028
Male	68 (57)	8 (32)	
Female	51 (43)	17 (68)	
ECOG-PS, No. (%)			.007
0	75 (63)	23 (92)	
1-2	41 (37)	2 (8)	
Primary site, No. (%)			.085
Stomach	35 (29)	15 (60)	
Small intestine	40 (34)	6 (24)	
Colon	5 (4)	0	
Rectum	2 (2)	0	
Unknown	37 (31)	4 (16)	
Clinical staging, No. (%)			.645
Recurrent	40 (34)	10 (40)	
Unresectable	80 (67)	15 (60)	
Metastatic organ, No. (%)			
Liver	69 (58)	8 (32)	.026
Peritoneum	68 (57)	7 (28)	.009
Lymph node	8 (7)	6 (24)	.017
Lung/pleura	7 (6)	1 (4)	1.000
Bone	6 (5)	0	.590
CGP test platform, No. (%)			.143
NCC Oncopanel	5 (4)	3 (12)	
F1	114 (96)	22 (88)	
Sampling site, No. (%)			.382
Primary site	54 (45)	14 (56)	
Metastatic site	65 (55)	11 (44)	
Sampling timing, No. (%)			.500
Before imatinib	43 (36)	11 (44)	
After imatinib	76 (64)	14 (56)	

Abbreviations: CGP, comprehensive genomic profiling; ECOG-PS, Eastern Cooperative Oncology Group Performance Status; F1, FoundationOne CDx Cancer Genomic Profile; GISTs, GI stromal tumors; NCC Oncopanel, OncoGuide NCC Oncopanel System.
